# Acceptability and potential impact of delivering sexual health promotion information through social media and dating apps to MSM in England: a qualitative study

**DOI:** 10.1186/s12889-019-7558-7

**Published:** 2019-09-06

**Authors:** Joanna M. Kesten, Kaiseree Dias, Fiona Burns, Paul Crook, Alison Howarth, Catherine H. Mercer, Alison Rodger, Ian Simms, Isabel Oliver, Matthew Hickman, Gwenda Hughes, Peter Weatherburn

**Affiliations:** 1 The National Institute for Health Research Health Protection Research Unit on Evaluation of Interventions, University of Bristol, Bristol, UK; 2 The National Institute for Heatlh Research Collaboration for Leadership in Applied Health Research and Care West (NIHR CLAHRC West) at University Hospitals Bristol NHS Foundation Trust, Bristol, UK; 3 Population Health Sciences, Bristol Medical School, University of Bristol, Oakfield House, Oakfield Grove, Clifton, Bristol, BS8 2BN UK; 4 Institute for Global Health, University College London, London, UK; 5 Royal Free London NHS Foundation Trust, Pond St, Hampstead, London, NW3 2QG UK; 6 Field Service, National Infection Service, Public Health England, London, UK; 7 National Infection Service, Public Health England, London, UK; 8 The National Institute for Health Research HPRU in Blood Borne and Sexually Transmitted Infections, University College London, London, UK; 9 Sigma Research, Public Health, Environments and Society, London School of Hygiene and Tropical Medicine, 15-17 Tavistock Place, London, WC1H 9SH UK

**Keywords:** Qualitative research, Acceptability, Sexual health information, Men-who-have-sex-with-men (MSM), Dating apps, Social media

## Abstract

**Background:**

Increasing rates of sexually transmitted infections (STIs) in men-who-have-sex-with-men (MSM) in England is a pressing public health concern. Interventions targeting MSM, including information provision that effectively promotes sexual health, are needed. To support such intervention development, it is necessary to understand acceptable ways of delivering sexual health information. We explored the acceptability and potential uses and impacts of delivering sexual health information to MSM through social media and geosocial networking apps or dating apps.

**Methods:**

Semi-structured interviews were conducted in person or by telephone with 25 MSM resident in England recruited via dating apps and social media advertisements. Interviews explored sexual health information sources, perceptions and uses. Attitudes towards sexual health promotion through social media and dating apps were then discussed. The data were analysed using thematic analysis.

**Results:**

Sexual health information delivery through social media and dating apps was considered acceptable. Receiving information when browsing social media was viewed positively by most, as people have time to absorb information. In contrast, concerns were expressed that sharing or commenting on social media sexual health information may lead to judgements and discrimination. While social media reaches a high proportion of the population, dating apps can easily target MSM. However, tensions exist between the ability to provide information at an opportune time through dating apps, when users are connecting with new sexual partners, with the potential to adversely affect the app user’s experience. Hypothetical and actual uses and impacts of sexual health information ranged from no impact to reading information, sharing with peers, and increased awareness, to influencing healthcare-seeking, decision-making and risk-taking behaviours. Ensuring that information is engaging, positive in tone, not too clinical, focused on building social norms and delivered by trusted organisations were viewed as important for supporting its use.

**Conclusions:**

Overall, these findings support the development of new interventions that use dating apps and social media for sexual health promotion.

**Electronic supplementary material:**

The online version of this article (10.1186/s12889-019-7558-7) contains supplementary material, which is available to authorized users.

## Background

Sexually transmitted infections (STI) continue to rise among gay men, bisexual men and other men-who-have-sex-with-men (MSM) in the United Kingdom (UK) [1–3]. Meeting sexual partners through geosocial networking apps, referred to as ‘dating apps’ hereafter, and social media [4] influences the transmission of STIs by increasing social networks, facilitating rapid partner change, disassortative mixing and, reducing the time for epidemics to spread [5–10]. Indeed, Beymer and colleagues found that use of dating apps for meeting sexual partners increased the likelihood of MSM testing positive for chlamydia and gonorrhoea compared to meeting partners through in-person methods [7].

Despite awareness of HIV, MSM knowledge of other STIs such as the prevalence, transmission route, health implications and treatment procedures is variable and often poor [11]. Effective public health interventions targeting MSM, including the provision of sexual health information on infection risks and symptoms, infection outbreaks, sexual health testing, treatment and where to find it, are therefore needed.

Health-related information delivered via the internet is inexpensive, widely accessible and allows users to remain anonymous [12, 13]. Social media (e.g. Facebook, Twitter) use, particularly among young people [14], is common and may offer a useful means to reach MSM; particularly those who do not identify as gay and do not access Lesbian, Gay, Bisexual, Trans, and Questioning (LGBTQ) services or sexual health services [4]. Social media interventions can be scaled up inexpensively [4] and offer a potentially effective mechanism for promoting safer sexual practices (e.g. condom use) [14, 15]. Interventions delivered via social media have aimed to prevent sexual risk behaviour [14, 15] and have been designed to increase pre-exposure prophylaxis (PrEP) uptake among MSM [4].

Dating apps are also used by many MSM [16, 17] and can support the tailoring of sexual health information to user location [16, 18]. Currently, most dating apps do not routinely provide or sign-post to sexual health information [19] but app-delivered interventions encouraging HIV/STI testing have been shown to be feasible and acceptable to MSM [16, 18]. However, concern about the privacy of information provision through these apps has been raised [20].

Therefore, while social media and dating apps play a central role in the transmission of STIs, they also offer potential intervention settings to deliver time-limited, brief (written) interventions, and promote access to other sexual health information and to increase precautionary behaviour and / or reduce risk behaviour [8].

There is currently a dearth of qualitative evidence exploring the acceptability and potential impact of sexual health information delivery through social media and dating apps. An understanding of the most acceptable and effective approach for delivering sexual health information is needed to inform, prioritise and support the effectiveness [21] of future interventions to reverse the trend in STIs in MSM. In this study, we explored the acceptability and potential impacts of delivering sexual health information to MSM through social media and dating apps.

## Methods

### Sampling and recruitment

England resident men or transgender men aged 16 years or over who had ever had sex or intended to have sex with a man, were recruited through adverts on the dating apps - Scruff (https://www.scruff.com) and Growlr (www.growlrapp.com). Participants were also recruited through advertisements on the research team’s twitter account and other relevant social media (e.g. OutBristol). An advert describing the study and inviting those interested to contact the researcher (JK) for more information on Scruff targeted Greater London and ‘Shout-outs’ (direct messages sent to users within a set radius of central postcodes in Bristol and Manchester), were issued through Growlr. These locations were chosen to ensure geographical variation.

JK confirmed eligibility with individuals responding to advertisements and emailed information sheets to those meeting the inclusion criteria.

### Interview organisation

A convenient date and time for participants was arranged to conduct semi-structured interviews, recorded using encrypted digital audio-recorders. For participants living in Bristol, face-to-face or telephone interviews were offered whereas participants outside of Bristol were only offered telephone interviews. Informed consent was obtained from all participants. Verbal informed consent was audio recorded for telephone interviews for practicality reasons and written informed consent was obtained prior to face-to-face interviews. We aimed to conduct interviews until theoretical saturation of emerging concepts was achieved. Participants received a £20 high-street shopping voucher in recognition of their time and effort.

### Topic guide

The interview topic guide (Additional file 1) was developed for this study and applied flexibly to allow emergence of unexpected issues. It explored sources of sexual health information, perceptions and uses of information, awareness of local STI outbreaks and acceptable means of health promotion messaging and attitudes towards the use of social media (e.g. Facebook, Twitter, Instagram) and dating apps to target MSM. The latter is the focus of the current paper. Participants were also asked background questions on their demographic characteristics such as age, ethnicity, education status and STI and HIV testing practices. Following the initial six interviews, the topic guide was adjusted to improve clarity of the questions and reflect emerging issues.

### Analysis

Audio files were transcribed verbatim and analysed using a data-driven, inductive thematic approach [19]. This method is suitable for qualitative research with clear aims and facilitates the elicitation of unexpected themes.

Interview 11’s audio file was accidentally deleted prior to transcription, fieldnotes taken during this interview were analysed instead.

Transcripts were repeatedly read by JK to gain familiarity with the data. JK then assigned codes systematically, line-by-line. Although coding was performed inductively, the codes were informed by the topic guide. The study team [IO, GH, PC, PW, FB, IS] discussed initial coding of six transcripts and these were then iteratively refined and combined to produce an agreed coding framework. After 15 transcripts were coded, PW and JK discussed in detail the coding and interpretation (summarised in descriptive accounts), of these transcripts to enhance the trustworthiness and rigour of the analysis by widening JK’s perspective. PW also reviewed three transcripts in detail to inform his understanding of the coding framework. Discrepancies in interpretation were resolved through discussion which helped further develop the analysis. The coding framework was refined and applied to all transcripts by JK as data emerged from subsequent interviews and as the analysis developed.

### Ethical approval

Ethical approval was granted by the University of Bristol, Faculty of Health Sciences Research Ethics Committee (Ref: 55961). The ethics committee approved the use of verbal informed consent procedures for telephone interviews.

## Results

Forty MSM responded to the advertisements on Facebook (*n* = 9), Growlr (*n* = 21), Scruff (*n* = 8) and through an unknown source (n = 2). After receiving the study information, one person declined to participate, four did not respond and 10 responded after theoretical saturation had been achieved and data collection had finished. In total, 25 MSM (Table 1) participated in an interview which lasted 43 min on average (range 26–57). Participants were most commonly 30–39 years (*n* = 10), white British (*n* = 19), had achieved a first or higher University degree (*n* = 22) and were full-time employed (*n* = 13). Two identified as trans male. Twelve participants were recruited from Growlr, 3 from Scruff, 9 from Facebook and, 1 from an unknown source.

**Table 1:** Participant Characteristics

Participant characteristics	n
*Recruitment source*	
Facebook	9
Growlr	12
Scruff	3
Unknown	1
*Age*	
20–29	8
30–39	10
40–49	4
50 and over	3
*Ethnicity*	
Asian British	1
Mixed race British	1
White British	19
White Irish	1
White Other	3
*Current residence*	
South West of England Bristol	13
South East of England	5
North West of England	6
North East of England	1
*Highest level of education*	
GCSE’s	1
A level	2
First degree	10
Higher degree (MSc, PhD)	12
*Gender*	
Male/cisgender male	23
Transgender masculine /	1
Transgender queer	1
*Employment status*	
Unemployed	4
Retired	1
Student	4
Self-employed	2
Part-time employed	1
Full-time employed	13
*Ever tested for STI*	
Yes	25
Total	25

The views of MSM did not substantially differ according to geographical location or recruitment source.

### Uses and impacts of sexual health information

This section does not specifically relate to information proactively received via dating apps or social media, experience of which was insufficiently common to discuss the actual utility or impact of information previously encountered online.

Hypothetical and actual *uses* and *impacts* of sexual health information received, ranged from no impact to reading information, sharing with peers, and increased awareness, to influencing healthcare-seeking, decision-making and risk-taking behaviours.

Information may be ignored and have no impact. Barriers to using sexual health information include a lack of concern or willingness to consider sexual health. One participant with HIV described low self-esteem prior to his diagnosis as limiting his receptiveness to information.

*It’s not just about having the information. We know that information is there and we ignore it or just don’t want to see it (…*). *There are times where I may take some risks and deep inside I do know what the risks are and I know there is information about risks online but in that moment, that’s not effective for me (…*). *So that’s the limitation but I can’t think of any issues with the information that find I online or elsewhere, but it’s more the approach sometimes, the information approach, that I think isn’t sufficient.* Interview 6, 32 years.

Information could reassure or increase anxiety about the seriousness of infections and their symptoms. It could inform lay diagnosis, and influence decisions on whether to seek medical help for some; either encouraging help-seeking or reassuring that this is not necessary. Conversely, for those who are comfortable seeking medical help, information may not be sought beforehand.

*I would look on a website, find out, “Well, yeah, actually by the sounds of it, it probably could be this,” and then access the health services so I could speak to a professional who could confirm one way or the other.* Interview 18, 46 years.

Provision of sexual health information was seen as a way to potentially increase STI and HIV testing, vaccinations and checking whether partners have been tested. The influence on sexual risk-taking was commented on hypothetically by some.

*Either it does or it doesn’t [influence behaviour]. It’s really difficult to say, isn’t it? There’s so much information out there these days that some information influences your behaviour and some information doesn’t. And often it’s about an aggregate of information influencing your behaviour rather than it’s one particular thing.* Interview 17, 39 years.

*Actually, when they [HIV campaign messages received on Grindr] first popped up saying ‘Know your status’ it reminded me that I hadn’t been tested in a while, so it actually prompted me to go and get a little blood test.* Interview 22, 32 years.

Information trustworthiness, reliability, personal relevance, circumstances/timing in which the information is received, and originality were highlighted as informing its utility. For example, information encouraging STI testing is unlikely to result in more frequent testing among those who already test regularly. Ensuring information is engaging, sex positive in tone, not too clinical and focused on building positive sexual health norms were viewed as important.

*I think it’s a good idea [for healthcare organisations to use social media or dating apps to share sexual health information] and I think it’s important to do so because by sharing that information from reputable sources people are more likely to trust the information that’s been given to them and also people are more likely to say, ‘oh, because I’ve had this information from these organisations, actually I’m more likely to get tested’.*

Interview 1, 34 years.

*There was a whole ‘It starts with me’ kind of campaign [designed to encourage HIV testing]. I remember that standing out and me not feeling I particularly needed to respond to that ‘cos I’m like ‘Well, I already do this [test regularly for HIV].’*

Interview 17, 39 years.

*I think it’s getting better but I think it’s taking the medical element out of it and actually speaking to people in the language that they speak (…*), *in quite a neutral tone rather than perhaps a tone that might make people scared or anxious about what they may or may not have.*

Interview 22, 32 years.

### Attitudes towards sexual health information provision via generic social media

Most MSM responded positively to the idea of healthcare organisations providing sexual health information through social media; a commonly used source of information and news. The timing of receiving information when browsing social media was viewed positively by most, as people have time to absorb information discreetly.

*It is in a (…*) *situation where people are just there and receptive to information but not actively looking for anything. If you’re scrolling through Instagram you’re just having a little browse.* Interview 9, 26 years.

*I think it’s the thing of not having to seek out that information, it’s being given to people. It can be given in such an easy way to such a wide reach of people and it makes it a lot more accessible, especially for people who have access needs. It might not be easy for them to get to a clinic or speak to somebody about it, if they can see that out on social media it would make it a lot easier.* Interview 25, 22 years.

Some participants felt that social media adverts in general were annoying and that targeted information, for example according to sexual orientation, could be creepy, intrusive and give the impression of being tracked. However, others commented that adverts were an accepted part of social media use and that targeting is a legitimate use of personal information.

*When I saw the ‘get tested’ ad, well I thought it was great and it was, (…*) *but at the same time I feel a bit tracked, like why am I getting this on Instagram? It was okay because at the end I did the test and everything – it was a new thing that I found. I learned that it worked and it helped me but, at the same time, I couldn’t avoid feeling a bit tracked or targeted.* Interview 7, 27 years.

Some participants did not see any negative consequences of providing sexual health information through social media. One participant highlighted that social media can offer peer support and only one participant highlighted internet access as a barrier. Social media was expected to reach younger MSM, and one participant reflected that it is important to ensure information is age appropriate. One participant commented that information received through social media may be less trusted, depending on the information source. A few participants highlighted concerns that sharing sexual health information via social media may lead to anxiety and paranoia, emphasising that the information should be posted discreetly and should not appear on newsfeeds for others to see. This was especially concerning if sexual orientation was undisclosed. There was some concern that sharing or commenting on social media sexual health information may lead to judgements, labelling and discrimination. Also, some were concerned the intended meaning of the information could be altered through posts which share and comment on it.

*Mostly gay people (…*) *are not out, (…*) *and they have their family and friends and colleagues on Facebook. It’s the same with me, so I would not like it. If I liked the page, then they will be appearing on my page and so I would not prefer that.* Interview 24, 33 years.

### Attitudes towards sexual health information provision via dating apps

Most MSM approved of healthcare organisations sharing sexual health information through dating apps via adverts, online chats, and signposting to further information on websites. For example, some participants talked positively about organisations such as the Terrence Higgins Trust using dating apps to provide advice and information directly to individuals through instant messaging conversations with users.

*The HIV one was through GROWLr. It just said, “If you answer these questions, you will be given a free HIV test.” Now, I have no reason to have any thoughts that I would have HIV. I just thought “That’s a useful thing to do.”* Interview 19, 58 years.

*Literally I need a pop up that comes up every five minutes just saying ‘be safe’!* Interview 2, 22 years.

Some participants described apps indirectly supporting information provision through an increasing trend of dating app users displaying their STI, HIV and PrEP status on their profile. While some disliked this feature, others appreciated the openness and were prompted to seek more information.

*You read on GROWLr, on people’s things, STI tested March 2018. So I thought “That’s quite a nice badge to have.”* Interview 19, 58 years.

Dating app information provision was perceived to reach the target audience and have the potential to act as a reminder of safe sexual practices at an opportune time – when people are intending to have sex. It enables information to be brought to people who may be too embarrassed to look for it themselves. Similarly, dating apps overcome the barrier of reaching people who are not actively seeking information or regularly accessing sexual health or LGBTQ services. For example, we found that older, more sexually experienced MSM who had decided their approach to sexual risk-taking and those in relationships tended to feel they did not need information as much as when they were younger or compared to other younger, less sexually experienced MSM.

*People are aware of STDs and what they are at my age you know and we kind of know what to do and we know where to go but most of that is from my 20’s.* Interview 13, 37 years.

*It’s such an easy way to specifically target gay men, or men-who-have-sex-with-men at least, and a lot of conversations on those apps will revolve around arranging sex and that kind of thing but to have the information there while you’re also having those conversations makes it more – it puts it in your brain more prominently.* Interview 25, 22 years.

Some participants queried whether promoting sexual health information may contradict the mission of the apps, but it was perceived to enhance the company’s credibility.

If sexual health messages were tailored to the user’s profile or content of conversations (e.g. prompted by use of key words in messages) this may enhance the personal relevance. However, some viewed this as intrusive and disturbing. A small number of participants commented that promoting sexual health information through dating apps may negatively affect the user experience, taking the “fun” out of meeting sexual partners, creating associations between meeting partners and infection risk and causing users to question whether the timing of adverts was related to the person they were talking to.

The timing of information provision, when people are looking for sexual partners, may also mean users are less receptive to the information. Interviewees also anticipated some annoyance from older, more sexually experienced, knowledgeable MSM who do not feel they need information. Concerns about discretion of information were raised by a small number.

*I think sometimes all that kind of information it does kind of take away like the pleasure of like sex and (…*), *hooking up with people or meeting people I suppose ‘cause I don’t know there’s always a small part of you just wanna go out and have fun.* Interview 20, 24 years.

*There may be some people who are a lot more experienced that just think, “Oh, God, they’re throwing this at us again. We know what to do” and something (−) A bit like an air hostess giving the instructions what to do if there’s a crash.* Interview 19, 58 years.

Pop-up adverts on dating apps were common and generally disliked but some were indifferent to pop-ups as they were easily ignored. In some apps, advertisements can be avoided by paying for a premium account. Some participants preferred banner style messages rather than pop-up’s as they were more discreet, less annoying and present less impedance to use. However, the depth of information which can be provided in banner messages is limited.

*For me, personally, because (…*) *I feel like I’m on top of my sexual health, I feel like it’s [pop-up adverts] a little bit of an annoyance. It’s a little bit irritating because I’ve probably gone to try and hook up with a guy, not to think about having sexual health check-ups, so I think it’s hard. I think they’re a good thing because they’re in a place where people are thinking about that, but at the same time (…*), *from a practical point of view, they are maybe a bit of an annoyance and they do get in the way.*

Interview 4, 41 years.

*Sometimes they do have little banner ones [adverts] at the bottom I guess which are a bit more discrete; but quite often they’re quite in your face and that can be quite frustrating and probably I would suggest that if you’re spreading like sexual health messages then actually frustrating users is probably not an ideal way to do it.*

Interview 3, 31 years.

Some participants felt that dating apps can be used to directly target MSM more easily than social media. Participants felt that because social media reaches a wider population than dating apps, the language should be less explicit or sexualised. Compared to dating apps, more information could be provided directly on social media due to the space available and because the information conflicts less with the purpose of use; finding sexual partners.

*People are probably more receptive to reading information when they’re on social media, as opposed to a dating app. Although, when they’re on a dating app, it’s much easier to target the right kinds of people that you would want to give that kind of information because everyone there is thinking about engaging in sexual behaviour or sexual encounters.*

Interview 9, 26 years.

*People prefer a positive tone on generalised social media, but you can have a little bit more information initially and it can be a bit more straightforward because again you’re not necessarily in a fight with people’s motivations. People are often just going on because that’s what they’re doing rather than they have a particular thing that they’re looking for.*

Interview 17, 39 years.

*On social media, don’t make it very sexualised, the wording, but in dating apps you can do that because that’s what the apps are about.*

Interview 12, 45 years.

## Discussion

### Summary of findings

To the authors’ knowledge this is the first qualitative study in England exploring the acceptability of sexual health information provision through social media and dating apps to MSM. Sexual health information delivery through these channels, from trustworthy, reliable sources was viewed as acceptable suggesting that these channels could be capitalised on to deliver messaging to MSM around sexual health. Diverse perceived uses and impacts of such information were captured ranging from no impact, to influencing healthcare-seeking, decision-making and risk-taking behaviours.

### Comparison to existing literature

There is agreement that efforts to raise awareness of STIs among MSM are needed [11, 22] and given the important role of dating apps and social media in the transmission of STIs they may offer a useful setting for intervention delivery [5–10].

This study’s findings corroborate research which suggests that social media and dating apps are likely to be acceptable platforms to deliver sexual health information [16, 18] and that both are feasible ways to reach MSM [14, 15]. In particular, these routes have the advantage of communicating with those who may not access sexual health services [4]. In addition, we identified nuanced responses, including potential negative effects of social media and dating app use for information provision. For example, the potential for anxiety from information sharing on social media means that information needs to be discreet to access and the importance of not hindering the user experience and pleasure of meeting sexual partners was key to the acceptability of information sharing through dating apps. In contrast, the timeliness of information provision through dating apps when people are considering meeting sexual partners and the receptivity to absorbing information when using social media were emphasised.

### Implications of our findings

Our findings suggest that social media and dating apps should be used more for health promotion as they appear to be acceptable ways to reach MSM. Ensuring such information is engaging, sex positive in tone, not too clinical, focused on building precautionary social norms and delivered by trusted organisations is also important. The benefit of using dating apps to deliver sexual health information is the ability to tailor information based on the individual’s geolocating features such as referring users to local HIV/STI testing services [16, 18]. However, personalisation or targeted information is a double-edged sword. People want information that is pertinent to them but find it intrusive to receive information which appears too precisely targeted to them.

There are specific sexual health messages that are important to disseminate to MSM regularly, and to younger MSM, in particular, who are becoming sexually active and newly accessing the gay scene. Proactive information provision through dating apps and social media, tailored to the user may also help encourage re-engagement among more experienced MSM.

### Strengths and limitations

This qualitative study has gained new, in-depth insights into the perspectives of MSM including nuanced responses to the use of social media and dating apps for sexual health information provision. We continued data collection until the same issues began to arise repeatedly and little new information emerged, therefore we are confident that theoretical saturation was achieved. However, by recruiting via dating apps and social media, our sample may be more likely to perceive this type of information channel positively. Experience of receiving information proactively via dating apps or social media was insufficiently common to discuss the utility or impact of information actually encountered online via apps or social media. Therefore, we were only able to capture hypothetical acceptability of the principle of using dating apps and social media to deliver sexual health information rather than gaining feedback on actual intervention content or delivery, or actual responses and impacts of such interventions. Furthermore, this method of communication may be limited by the potential for it to be presented alongside information that facilitates potentially high risk sexual contexts such as public sex environments.

The self-selected sample, which was not purposively recruited, reflected a range of ages and perceived sexual experience; however, we did not achieve diversity in relation to socio-economic status or ethnicity and all participants had tested for STIs. In addition, the majority of participants were from the south west of England. Therefore, the sample and the views expressed may not reflect all MSM.

## Conclusions

Overall, these findings support the use of dating apps and social media for sexual health promotion aimed at reducing STIs among MSM. More research is needed to develop interventions using these platforms and to evaluate the specific impact of such health promotion activities.

## Additional file

Additional file 1: Sexual health information for men-who-have-sex-with-men (MSM) project interview topic guide. Semi-structured interview topic guide. (DOCX 33 kb)

## Data Availability

Data are available at the University of Bristol data repository, data.bris, at 10.5523/bris.2wcebrf0io8wx2hbdxlv0wpmkf. Data access is restricted to bona fide researchers for ethically approved research and subject to approval by the University’s Data Access Committee.

## Abbreviations

LGBTQ: Lesbian gay bisexual transgender questioning
MSM: Men-who-have-sex-with-men
PrEP: Pre-exposure prophylaxis
STI: Sexually transmitted infection
