# Supplementary material for: Acceptability and potential impact of delivering sexual health promotion information through social media and dating apps to MSM in England: a qualitative study
Source: BMC Public Health. 2019 Sep 6;19:1236. doi: 10.1186/s12889-019-7558-7 (PMC6728978; doi:10.1186/s12889-019-7558-7)
Supplement: Supplementary file 1 — Sexual health information for men-who-have-sex-with-men (MSM) project interview topic guide. Semi-structured interview topic guide. (DOCX 33 kb) [file 12889_2019_7558_MOESM1_ESM.docx]

**Additional files**

**Additional file 1**

**Sexual health information for men-who-have-sex-with-men (MSM) project**

**Interview Topic Guide**

***Equipment***

- Audio-recorder and spare batteries
- Refreshments (if face-to-face interview)
- Incentive (to be sent by post if telephone interview)

***Introduction***

- **Introduce researcher.**
- **Thank you very much for sparing the time to speak to me today.**
- **Just to give a background to the study, there is a need to research suitable, acceptable and effective approaches to deliver sexual health information to men who have sex with men.**
- **Sexual health information can be defined as information on infection risks and symptoms, infection outbreaks, sexual health testing, treatment and where to find it.**
- **The interview will be audio-recorded. Anything that you tell me is confidential and will not be linked to you. You can stop at any time and if you decide in the next two weeks that you don’t want me to include what you said, just let me know.**

**Ask where heard about study if do not already know**

**Answer questions**

**Face-to-face interviews: Complete consent form**

**Commence audio recording**

**Telephone interviews: Consent**

As this is a telephone interview we need to take informed consent for your participation verbally. I am going to read six statements to you and I would like you to confirm whether you are happy with each statement by answering yes:

1. I confirm that I have read and understand the ‘Sexual health information for MSM Participant Information Sheet’ (version 1.0 dated 18/08/2017). I have had the opportunity to consider the information, ask questions and have had these answered satisfactorily.

2. I understand that the information I give will be kept strictly confidential and used only for the purposes of this study. My consent depends on the University of Bristol complying with its duties and obligations under the Data Protection Act.

3. I agree to my interview being audio recorded.

4. I agree to the study publishing anonymous quotations from the interviews and understand that it will not be possible to identify me in any way.

5. I understand that only members of the research team will have access to my personal data and that the overall anonymous data from the study may be seen and used by other researchers, for ethically approved research projects, on the understanding that confidentiality will be maintained.

6. I understand that regulatory authorities may require access to the study information where it is relevant to my taking part in this research. All information accessed will remain strictly confidential. I give permission for these individuals to access this information.

7. I understand that my participation is voluntary and that I am free to withdraw at any time, without giving reason, without my medical care or legal rights being affected.

8. I agree to take part in the above study.

***Questions***

**1. Sexual health information seeking**

There are three main topics I’d like to cover. Firstly, I’d like to start by talking about sexual health information which you may have actively looked for.

1. Do you ever look for sexual health information (e.g. safe sex options – condoms etc., Sexually Transmitted Infections, HIV and sex/relationships, outbreak information in general)?
2. What **type of information** do you look for? PROMPT: anything else…

*Probe:* STI signs and symptoms, HIV, infection transmission, location and contact details of sexual health centres, treatment for STIs, sex and relationship advice, chem-sex information, PrEP information

1. [If yes] **Where** do you ***currently*** look for or see sexual health information?

*Probe:* Sexual health centres, online search engines, friends, leaflets etc

1. **Where** have you looked for sexual health information ***in the past***?
2. What are your **reasons** for choosing these sources both past and present?
3. What **prompts** you to search for information about sexual health?

*Probe*: After a sexual encounter? After hearing about a friend’s experience? Out of interest? To inform decision about visiting sexual health clinic?

1. What is **good** about sexual health information which is currently available?

*Probe:* Easily accessible, free, plain language, available confidentially, information without judgement or stigma, MSM specific information etc

What is **not good** about sexual health information which is currently available?

*Probe:* Difficult to access, difficult to interpret, concern about lack of confidentiality, stigma, lack of MSM specific information etc.

How could the available information be **improved**?

Probe: e.g. Sexual health information questions asked on an online forum and answers posted online.

1. Is there any **information which you want but cannot easily access**?
2. [If not mentioned above] Would you prefer **more signposting** to information or to be provided with information?
3. Are there any **sources** where you would prefer to find sexual health information from where you do not currently access it? If so, where would you prefer information to be available?

*Probe:* Dating apps, schools, bars and nightclubs, TV etc.

1. Has sexual health information been provided through your wo**rkplace or higher education institution**?
2. What do you think about healthcare organisations like the NHS using websites to share sexual health information in general?

[IF NOT ALREADY ADDRESSED ABOVE]

1. What things [if any] **encourage** you to use **online sexual health information**?
2. What things [if any] **discourage** you to use **online sexual health information**?

*Probe:* e.g. seeking information through Facebook may be visible to friends and family

1. Are there any **negative consequences** from presenting sexual health information **online**? If, so what are the negative consequences?

*Probe:* Annoyance? Reluctance to use online platforms? Feel stigmatised? ‘Google-effect’ (believing you have a serious condition)?

**2. Acceptability of ‘push’ messages**

OK so now I’d like to move on to talk about sexual health information you have received or seen without having to actively look for.

1. Do you ever receive/see sexual health information without having to look for it? If so, from **where**?
2. What **type of sexual health information** do you receive in this way (i.e. without actively looking for it?)
3. What **type of information** would you like to receive in this way, but do not currently?

***‘Push’ messages about infection outbreaks***

1. Have you ever received information about outbreaks of infections in the local area? If so, how?
2. How do feel about the way this information is shared?
3. How would you ideally like to receive information about outbreaks?
4. Is there anything that can be done to improve the way this information is shared?

***‘Push’ messages through social media***

1. What sort of social media platforms do you use?

*Probe:* Facebook, Twitter, Snapchat etc

1. What do you think about adverts that pop up when you are using social media?

*Probes:* Do you think that they come up too often or too little?

1. What do you think about healthcare organisations like the NHS using social media to share sexual health information?

*Probe:* What do you like/dislike about this use of social media?

1. How/for what **purpose** do you/would you use sexual health information on **social media**?
2. Are there any **negative consequences** from presenting sexual health information on social media? If, so what are the negative consequences?

*Probe:* Annoyance? Reluctance to use online platforms? Feel stigmatised?

***‘Push’ messages through dating apps***

1. What sort of dating apps platforms (if any) do you use?

*Probe:* Grindr, SCRUFF, Growler etc

1. What do you think about **adverts** that pop up when you are using **dating websites**?

*Probes:* Do you think that they come up too often or too little?

1. What do you think about healthcare organisations like the NHS using **dating websites/apps** to **share sexual health information**?

*Probe:* What do you like/dislike about this use of social media?

1. How/for what **purpose** do you/would you use sexual health information on **social media**?
2. Are there any **negative consequences** from presenting sexual health information on dating apps? If, so what are the negative consequences?

*Probe:* Annoyance? Reluctance to use online platforms? Feel stigmatised?

1. Do you have a **preference** between receiving sexual health information (including outbreak information) from face-to-face, written materials, websites, social media and dating websites/app platforms? If so, what are your reasons for this preference?

**3. Consequences of information provision**

Finally, I’d like to talk about how you use sexual health information which you either look for or receive without actively looking for.

1. When you come across links to further information on social media or dating apps, what prompts you to click/not click on the link?
2. **How do you use/what do you do with sexual health information** (including outbreak information) you’ve found or been given?

*Probe:* decision-making re. help seeking behaviour, influence sexual behaviour, pass information on to others, share on social media

1. What things **influence** (encourage/discourage) whether you use information in this way?

*Probe:* setting information provided through, seeking vs receiving information?

1. How could you and other people be **supported/encouraged** to use sexual health information through websites, social media and dating apps? **How would these strategies differ for each?**

**Background questions**

The last few questions are to help us get an overview of the people we are speaking to.

1. How old are you (in years)?
2. What is your gender?

Male

Female

Trans male

Trans female

Other

Prefer not to say

1. Which of the following options best describes how you think of yourself? Heterosexual/Straight; Gay/Lesbian; Bisexual; Other
2. Where do you currently live?
3. What is your ethnicity?
   1. White British
   2. White Other
   3. Black British
   4. Black Caribbean
   5. Black African
   6. Asian British
   7. Indian
   8. Pakistani
   9. Bangladeshi
   10. Chinese
   11. Mixed (Please specify below)
   12. Other (Please specify below)
4. What is your current employment status?
   1. Student
   2. Househusband or Housewife
   3. Full-time (Employed or Self-employed)
   4. Part-time (Employed or Self-employed)
   5. Unemployed
5. What is your highest level of education?
6. No official qualification
7. Up to GCSEs / GCEs / 'O' Levels or equivalent
8. 'A' levels / NVQs / GNVQs or equivalent
9. First degree/ diploma/ HNC/ HND or equivalent
10. Higher degree (e.g. MSc, PhD) or equivalent
11. Have you ever been tested for sexually transmitted infections (STIs)?
12. Have you ever been tested for HIV specifically?
    1. Yes
       1. Where do you tend to get tested? STI clinic, online/postal etc.
       2. Ever tested positive? If so, what for?
       3. How frequently do you test?

***Closing***

- **That’s all the questions I have for you today. Do you have any questions for me?**
- **Thank you again for your time and help with this study I really appreciate you sharing your thoughts and opinions with me. Have a nice day.**
- **Give or send participant thank you gift for their time.**
